# Effects of spinal deformities on lung development in children: a review

**DOI:** 10.1186/s13018-023-03665-0

**Published:** 2023-03-27

**Authors:** Yonggang Wang, Dongmin Wang, Guangzhi Zhang, Bing Ma, Yingping Ma, Yong Yang, Shuai Xing, Xuewen Kang, Bingren Gao

**Affiliations:** 1 Department of Cardiac Surgery, Lanzhou University Second Hospital, No. 82 Cuiyingmen, Lanzhou, 730030 Gansu Province China; 2 Medical College of Northwest Minzu University, No. 1 Northwest Xincun, Lanzhou, 730030 Gansu Province China

**Keywords:** Early-onset scoliosis, Lung development, Spinal deformity, Thoracic deformity, Thoracic insufficiency syndrome

## Abstract

Scoliosis before the age of 5 years is referred to as early-onset scoliosis (EOS). While causes may vary, EOS can potentially affect respiratory function and lung development as children grow. Moreover, scoliosis can lead to thoracic insufficiency syndrome when aggravated or left untreated. Therefore, spinal thoracic deformities often require intervention in early childhood, and solving these problems requires new methods that include the means for both deformity correction and growth maintenance. Therapeutic strategies for preserving the growing spine and thorax include growth rods, vertically expandable titanium artificial ribs, MAGEC rods, braces and casts. The goals of any growth-promoting surgical strategy are to alter the natural history of cardiorespiratory development, limit the progression of underlying spondylarthrosis deformities and minimize negative changes in spondylothorax biomechanics due to the instrumental action of the implant. This review further elucidates EOS in terms of its aetiology, pathogenesis, pathology and treatment.

## Introduction

The growth of the lungs depends on the thoracic cavity, which includes the spine, ribs and sternum, particularly, whether sufficient space is available in terms of its height, width and depth [1–3]. A normal thorax is considered to have a normal and stable volume and the ability to change its volume. The ribs and sternum determine the depth and width of the thoracic volume, whereas the thoracic spine determines the vertical height [4]. In addition to achieving a stable volume, the thorax must also be able to change its volume, to maintain normal lung function, which depends on the coordinated active movement of the diaphragm and the intercostal muscles during breathing.

Campbell et al. [5] proposed the term ‘thoracic insufficiency syndrome (TIS)’ in 2003 to describe skeletally immature children and adolescents with respiratory and lung developmental disorders due to spinal and thoracic deformities, in which the chest cannot support normal breathing or lung growth [4]. There are complex interactions between spinal thoracic deformities, pulmonary parenchymal dysfunction and neuromuscular diseases that result in the impairment of ventilation pump function, which eventually leads to TIS. This syndrome may present itself in patients with prominent respiratory symptoms during physical examinations in which chest deformities, abnormal chest X-ray or computed tomography (CT) findings or changes in pulmonary function are revealed. In untreated EOS, progressive pulmonary hypofunction can lead to an increased risk of respiratory failure and early death [6]. Thus, one of the therapeutic goals of EOS is to restore lung function and lung capacity during growth.

The aetiology of TIS may be secondary to three separate pathological processes or a combination of them. The first condition can be caused by EOS, in which rotation of the spine results in torsion of the thorax that limits the volume of the thorax and movement of the associated ribs; this adversely affects thoracic growth and function, leading to the development of TIS. Notably, this condition is the most common [7]. The second condition is a generalized one in which all components of the chest (ribs, sternum and spine) are affected, e.g. thoracic dysplasia, rib dysplasia, osteogenesis imperfecta and achondroplasia [8]. The third condition involves neuromuscular dysfunction that affects the normal function of the chest, such as in static encephalopathy, muscular dystrophy, muscle atrophy, spina bifida or spinal cord injury [9]. All three of these conditions can cause scoliosis; thus, they are referred to as EOS. However, when EOS develops to the point where breathing is affected, it is then referred to as TIS.

Posterior spinal fusion is the traditional treatment for scoliosis, but it can be problematic in children who have not yet reached skeletal maturity, due to the need for continuous growth of the spine and thorax [10]. If the vertical growth of the spine is hindered, this tends to further exacerbate lung development problems by restricting the growth of the thorax. Karol et al. [11] reported that children with scoliosis (where primary lung disease, skeletal dysplasias or neuromuscular disease were excluded) have a high rates of severe restrictive lung disease after thoracic fusion before the age of 9 years. In addition, the amount of reduction in thoracic spine height is associated with smaller forced vital capacity (FVC), suggesting that more extensive fusion increases the risk of restrictive lung disease.

The purpose of this article was to review the literature on EOS and TIS over the last 20 years, in an attempt to provide a detailed description of the aetiology, pathophysiology, clinical evaluation and treatment options for TIS.

## Normal development of the thorax and lungs

Breast growth is complex and depends on an increase in the height of the thoracic spine, an increase in the symmetry of the rib cage and correct orientation of the ribs. Namely, the thoracic spine determines longitudinal changes in thoracic volume [12]. Growth and development of the lungs and alveoli accelerate at the same time as the growth of the spine, basically from birth to age 5 [13]. The thoracic spine will grow by 1.4 cm per year from age 0–5 years, 0.7 cm per year from age 6–10 years and 1.2 cm per year during puberty. The thoracic spine is 12 cm in length at birth, 18 cm at 5 years and over 27 cm in adulthood [14]. The latest research shows that if humans did not grow to their average height by the age of 5 years, then TIS onset would be likely [14]. Thus, the period from birth to age 5 years is considered the ‘golden period’ of lung and spine development [14, 15].

The width and depth of the rib cage are determined by the growth and orientation of the ribs. At birth, the ribs are oriented horizontally, so that as the length of the ribs increases, the diameter of the ribcage increases, and a square cross section develops. However, by the age of 2 years, the orientation of the ribs changes, causing the ribs to slope downward and the thorax to be oval in cross section. Thus, the cross-sectional volume of the rib cage depends on the growth of rib length and the degree of rib inclination. However, excessive downward tilt flattens the chest, reducing the sagittal depth and thoracic volume [16]. At birth, the thoracic volume is 6.7% of the adult thoracic volume. The thoracic volume increases to 30% and 50% of the adult volume by age 5 and 10, respectively. From age 10 to skeletal maturity, the breast volume grows the fastest, doubling in size, such that a rectangular thoracic cross section is eventually seen [12].

Lung volume increases 30-fold from birth to lung maturity [9]. At the airway level, only an increase in volume is involved, up to twofold to threefold. The numbers of capillaries and alveoli increase as well. Specifically, as the number of alveoli doubles, the surface area of alveoli and capillaries increases tenfold [14]. About 85% of the alveoli are formed after birth, and most of them are present by the age of 2. Several studies have shown that humans do not grow new alveoli after the age of 7 years [14]. However, recent magnetic resonance imaging (MRI)-based research revealed that alveolar development may occur throughout adolescence [17].

Lung development also depends on the expansion of the chest cavity. At birth, the expansion of the chest cavity is sevenfold that of the lungs and declines gradually each year [9]. Therefore, lung and thoracic growth are directly related [18], and diseases involving the thorax and thoracic vertebrae may adversely affect morphological changes in the lungs, which, in turn, are directly related to respiratory function during development.

## EOS aetiology and classification

In their original study, Campbell et al. classified TIS into two categories [3, 4].The first category includes deformity of the thoracic cavity caused by spinal deformity. Here, secondary thoracic deformity is caused by primary thoracic dysplasia or neuromuscular dysfunction, which can impair thoracic volume and thoracic cavity function; this is referred to as primary TIS [3, 4, 19]. The second TIS category, referred to as secondary TIS, describes conditions in which the entire spine abnormally affects diaphragmatic motor mechanics, thereby limiting resting lung volume and ventilation, as in paralytic progressive neuromuscular scoliosis [3, 20]. Progression of spinal thoracic deformity with age can lead to severe restrictive lung disease, respiratory failure and early death [3, 4, 20, 21].

**Fig. 1:**
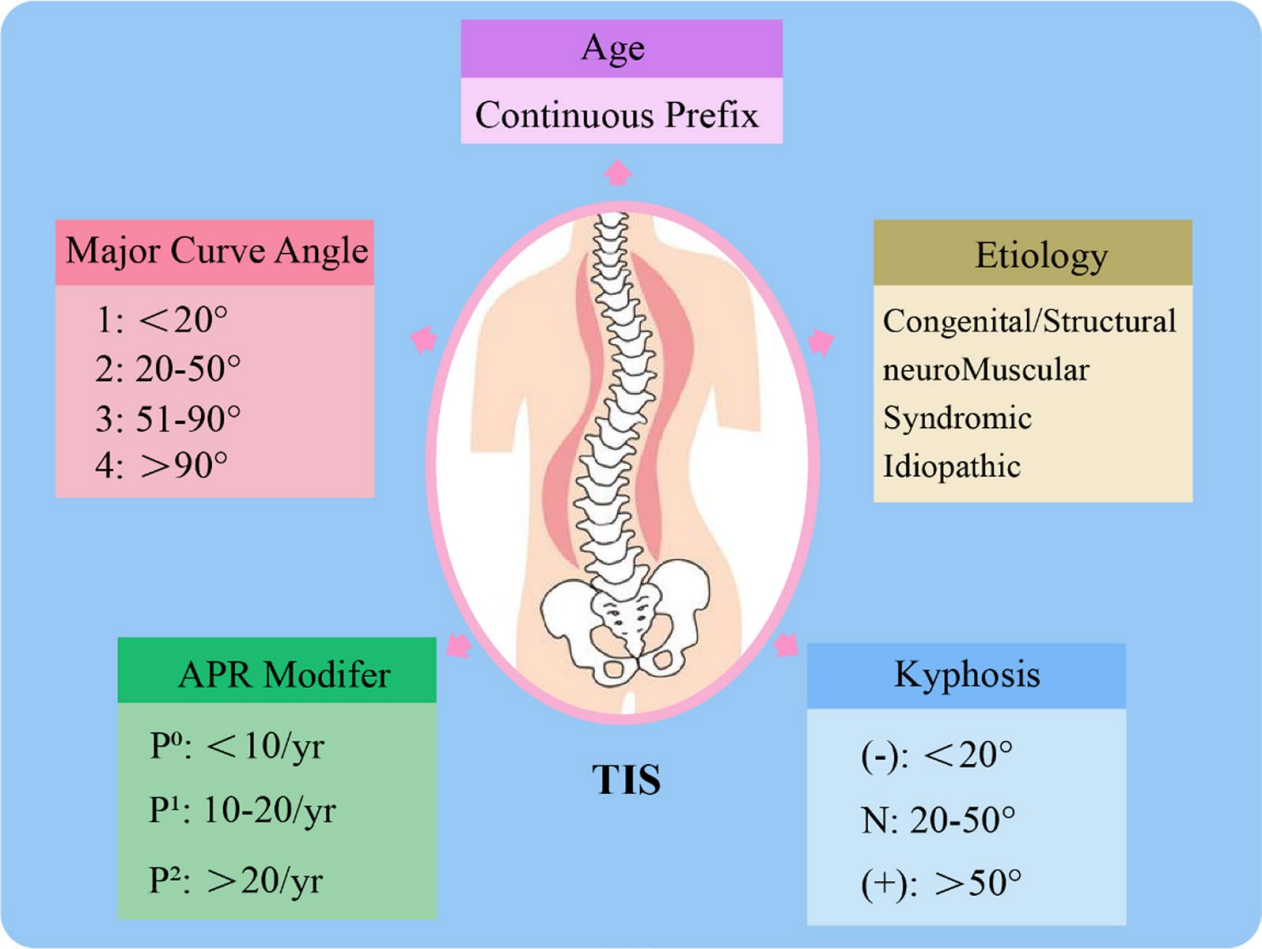
Classification of early-onset scoliosis (EOS). APR = annual progression ratio; TIS = thoracic insufficiency syndrome

**Fig. 2:**
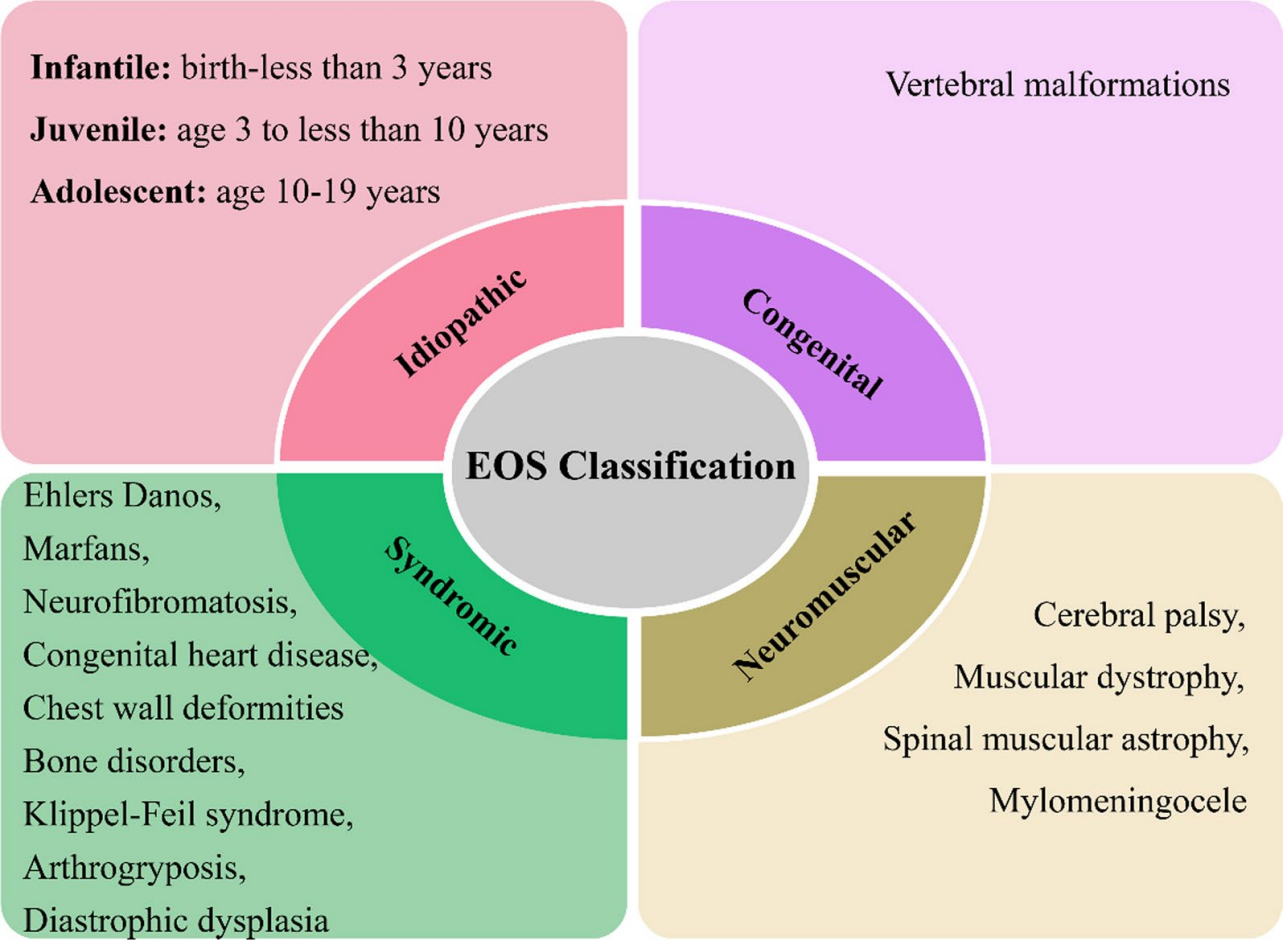
Aetiological classification of EOS. EOS = early-onset scoliosis

EOS is a complex disorder with diverse manifestations and natural histories. Any spinal deformity detected before the age of 5 years is considered an EOS case, with the patient at increased risk for progression and complications secondary to residual growth [22]. EOS may involve the development of breathing problems with age, thus indicating TIS. Diseases that affect the thorax and spine can cause deformity of the spine. As such, Williams et al. [23] classified EOS in terms of the curve angle (Fig. 1); TIS is then determined from the aetiological classification of EOS (Fig. 2).

### Idiopathic scoliosis

Idiopathic scoliosis (IS) occurs in 1% to 3% of people with scoliosis. It is more prevalent in girls and is classified according to the underlying cause or related disorder [24, 25]. The most common manifestations are right-sided curvature of the thoracic spine and left-sided curvature of the lumbar spine, resulting in S-shaped scoliosis; however, the condition can present itself in other forms. A London scholar has found in the histochemical examination and physiological study of paravertebral muscles in 17 patients with idiopathic scoliosis [26]. Histochemical tests of paravertebral muscles showed that it had universal aerobic metabolic potential, and there was no evidence of myopathic changes. Physiological studies showed that scoliosis was an organic disease rather than a systemic disease. In the absence of treatment, persistent scoliosis progression can lead to chest wall deformities such as rib rotation, resulting in restrictive lung disease, worsening lung function, reduced FVC and total lung capacity [27].

### Congenital scoliosis

Congenital scoliosis (CS) is caused by abnormal vertebral development during the embryonic stage (4–6 weeks of gestation), such as failure of vertebral segmentation (resulting in a ‘striped’ vertebral body) or when part or all of the vertebral body exhibits failure of vertebral formation (half vertebral body, butterfly-shaped vertebral body or wedge-shaped vertebral body) [28]. These abnormalities can cause mild to severe spinal deformities, depending on their combination and extent. CS often accompanies other congenital anomalies and is a component of multiple organ syndrome [29]. CS with rib fusion is a rare, but potentially life-threatening disorder that typically results in a small and stiff thorax. Children with this disorder may experience respiratory failure, pulmonary hypertension, right ventricular failure and cor pulmonale or death [30].

### Neuromuscular scoliosis

Neuromuscular scoliosis (NS) is a type of scoliosis that occurs in children due to progressive or non-progressive paralysis, weakness and/or disturbed muscle function of the spinal column [31, 32]. The NS scoliosis is often associated with central or peripheral nerve and muscle diseases [33]. NS usually presents as a longer, C-shaped curvature. This type of scoliosis is usually secondary to cerebral palsy, spinal cord injury, muscular dystrophy and other neuromuscular disorders.

#### Cerebral palsy

Cerebral palsy (CP) is defined as a permanent, non-progressive damage to central nervous system that results in abnormal motor function. This damage to the developing brain can occur during pregnancy or the perinatal or postpartum period for a variety of reasons [34]. CP is closely linked to the development of scoliosis. It is estimated that 21–64% of CP patients develop scoliosis [35]. Compared to IS, scoliosis in children with CP tends to begin at an earlier age and progresses more rapidly and beyond skeletal maturity resulting in more severe deformity. The deformity of the thorax leads to ventilation and diffusion dysfunction; as well as poor coordination of respiratory muscles and respiratory assist muscles, which gradually makes breathing shallower and less efficient; and excessive negative pressure in the upper and middle airways leads to narrowing of the airway, increased airway resistance, reduced ventilation and decreased lung compliance. This eventually leads to TIS.

#### Spinal muscular atrophy

Spinal muscular atrophy (SMA) is an autosomal recessive disorder caused by the deletion of the SMN1 gene with a prevalence of approximately 1/100,000 to 2/100,000. In general, SMA is characterized by a loss of cells in the anterior horn of the spinal cord, resulting in muscle weakness and atrophy. The progressive weakness of trunk/back muscles while vertebral column continues to grow against gravity, leads to development of scoliosis, pelvic obliquity and hip subluxation [36, 37]. More than 90% of children with type I or type II SMA will develop scoliosis [38]. The development of scoliosis involving thoracolumbar curve further limits the expansion of chest cavity during respiratory movements. In severe cases, it can be accompanied by significant lung failure, eventually leading to death. However, advances in modern medicine have increased the life expectancy of these patients [39].

#### Muscular dystrophy

Muscular dystrophy (MD) is caused by a deficiency of dystrophin in skeletal muscle, which results in muscle fibrosteatosis [40]. MD leads to scoliosis in 90% of boys; however, scoliosis development can be delayed into early adulthood with early treatment with steroids [41]. Affected infants typically present with weakness, low muscle tone, poor spontaneous movements, delayed or stalled gross motor development and associated stiffness of the joints and spine. Respiratory damage may affect the lifespan [42].

## Syndromic scoliosis

### Ehlers–Danlos syndrome

Ehlers–Danlos syndrome (EDS), an inherited connective tissue disorder primarily identified as multipoint hypermobility, is associated with musculoskeletal manifestations, lesser skin involvement and symptoms of chronic pain, chronic fatigue, autonomic impairment and anxiety [43]. In EDS, development of scoliosis is secondary to muscle hypotonia and ligament laxity and reported in more than 50% of EDS patients [44, 45]. The scoliosis may continue to develop and progress beyond puberty. However, most patients do not require intervention.

### Marfan’s syndrome

Marfan’s syndrome is an autosomal dominant disorder caused by a heterozygous pathogenic variant in the FBN1 gene encoding febrile 1 [46]. Its clinical features include aortic root dilatation, mild valgus and characteristic skeletal disease. Several musculoskeletal manifestations of Marfan’s syndrome, specifically, scoliosis, chest wall deformity and lung involvement, are common features of the syndrome [47]. Development of scoliosis and chest wall contraction may result in TIS. With progressive rotation of the spine chest expansion and volume in further reduced, thus, internal lung problems are often present.

### Neurofibromatosis type 1

Neurofibromatosis type 1 (NF-1) is an autosomal dominant genetic disorder affecting multiple organ systems that is characterized by abnormalities in the neural tissue, bone, soft tissue and skin [48]. Scoliosis is the most common disease associated with NF-1, and there are two main types: dystrophic and non-dystrophic [49]. Dystrophic scoliosis is characterized by an early onset, rapid progression and difficulty in treatment. Non-dystrophic scoliosis behaves similarly to IS, is more common and is treated similarly. The pattern of pulmonary dysfunction in patients with NF-1-related scoliosis is similar to that in IS [50], with more severe pulmonary dysfunction in patients with thoracic scoliosis.

### Klippel–Feil syndrome

Klippel–Feil syndrome is characterized by congenital bony fusion of some or all of the cervical spine [51]. Congenital cervical fusion results from poor segmentation along the embryonic developmental axis during the first 3–8 weeks of pregnancy. Diagnosis is based on clinical trials revealing a short neck, a low posterior hairline and limited neck movement. Other abnormalities include rib and thoracolumbar deformities and heart defects [52]. Clinically associated with this syndrome are scoliosis and other thoracic deformities; cardiorespiratory dysfunction may also occur. Abnormal rib spacing and rib fusion, as well as cost-vertebral joint deformities, limit thoracic elevation and movement thereby impacting respiratory function.

### Arthrogryposis multiplex congenita

Arthrogryposis multiplex congenita (AMC), also known as multiple congenital contractures, is a group of non-heterogeneous disorders [53]. Spinal deformities usually present at birth, progress rapidly in infancy and remain stable in later childhood. Most scoliosis cases are detected by the age 5 years [54]. For patients with a nonspecific diagnosis of AMC (possibly a combination of amyotrophic and syndromic arthritis), thoracolumbar scoliosis is the most common, with lumbar scoliosis being less common [55]. Patients with distal joint dislocation can develop severe thoracolumbar scoliosis, which can compress the lung tissue on both sides, and the small airways in the lungs can become deformed and distorted, thereby impairing the patient’s lung function [56].

### Jarcho–Levin syndrome

Jarcho–Levin syndrome (JLS) is an autosomal recessive congenital skeletal disorder with no known [57]. It is characterized by abnormal segmentation of the thoracic vertebrae and irregular fusion of the ribs that lead to EOS, restrictive lung disease and ultimately, TIS [58]. JLS includes two distinct genetic disorders—spondychochondrogenic dysplasia (SCD) and spondylothoracic dysplasia, and presents with multiple vertebral and rib abnormalities [59]. SCD is characterized by various abnormalities associated with spine and rib development, as well as congenital vertebral defects [60]. Another characteristic finding is the presence of congenital malformations of the ribs, small thorax, marked shortening of the trunk, fusion of the ribs to create a scalloped appearance and fusion of the occipital bone of the skull with C1. Because of these abnormalities, patients with JLS are more likely to develop TIS, which may eventually lead to early neonatal death. Despite these serious complications, approximately 50% of cases survive into adulthood [61, 62].

### Jeune syndrome

Jeune syndrome (JS), also known as asphyxiating thoracic dystrophy, is a rare multisystem autosomal recessive congenital disorder characterized by skeletal dysplasia, severe thoracic hypoplasia, exogenous restrictive lung disease, progressive renal disease and varying degrees of liver, pancreas, kidney, lung disease and retinal abnormalities [63]. Although a small bell-shaped ribcage is the most prominent feature in these patients, breathing is almost entirely supported by the diaphragm. The small, narrow chest and short limbs of JS patients, as well as respiratory failure onset, result in high neonatal mortality [63].

### Fibrodysplasia ossificans progressiva

Fibrodysplasia ossificans progressiva is an extremely rare and disabling genetic disorder defined by congenital skeletal deformity and progressive heterotopic ossification [64]. It is characterized by massive heterotopic ossification of connective tissue that leads to progressive arthrodesis and severe disability [65]. The development of restrictive lung disease due to marked heterotopic ossification involving the chest wall, spine and thorax often results in cardiopulmonary insufficiency syndrome, pneumonia and, in severe cases, death [66]. TIS may first manifest itself as limited expansion of the chest, characterized by deformity of the rib cage with in situ ankylosis; ossification of the intercostal muscles, paraspinal muscles and aponeurotic nerves; and progressive spinal deformity that, in severe cases, presents as a rigid chest wall that is unable to expand, allowing for only diaphragmatic breathing [65, 67].

### Congenital diaphragmatic hernia

Congenital diaphragmatic hernia (CDH) is a severe congenital anomaly in which the formation of diaphragm tissue is incomplete during development and abdominal viscera migrate into the thoracic cavity [68]. CDH can occur before birth (CS) or develop later in childhood [69]. Foetal pulmonary hypoplasia and pulmonary hypertension in patients with CDH lead to high mortality rate [70]. Survivors with CDH may present with multiple comorbidities, including respiratory insufficiency, regurgitation, dysplasia, recurrent hernias and musculoskeletal deformities [71]. Scoliosis is one of the more common musculoskeletal deformities associated with CDH.

## Pathophysiology

The complete rib–vertebra–sternal complex creates a flexible three-dimensional (3D) cuboid cavity in the chest. In scoliosis, this cavity becomes flat and rigid, limiting the expansion of the lungs [72]. However, there is strong evidence indicating that interactions between the growth of the spine and the progression of spinal deformities can alter/influence the size and shape of the ribcage [73]. Respiratory failure is the leading cause of high mortality in untreated scoliosis, not only due to extrinsic impairment of chest wall function but also intrinsic factors related to alveolar dysplasia [74]. In recent years, the focus has shifted from the spine to consideration of the spine, chest wall and lungs in combination, as treatments for spinal thoracic deformities. This new assessment has significantly improved lung development and function [72]. Within 5 years of age, avoidance of proximal thoracic fusion and a T1-T12 height of at least 22 cm, those are considered key to avoiding restrictive lung disease [16, 75]. Additionally, researchers agree that lung growth is largely complete before the age of 8, and the ‘golden period’ of maximum growth occurs before the age of 5 [76]. Therefore, it is important to maintain chest growth and lung volume during this critical period.

Autopsy studies have also shown that mechanical compression of the lung caused by chest wall deformity, which is not a factor in the reduction in alveolar numbers, may be related to premature cessation of alveolar hyperplasia [77]. In an EOS rabbit model, established using surgical fusion of one rib at 6 weeks of age, a study found that the number of alveoli in adulthood was reduced and the structure simplified, suggesting that postnatal chest wall constraints may directly affect postnatal lung morphology and development [78].

Respiratory changes in TIS range from exercise-induced tachypnoea to life-threatening respiratory failure. The main features of TIS are thoracic contraction and deformation and primary or secondary respiratory muscle weakness. These abnormalities include decreased intrathoracic volume and chest compliance, which in turn reduces chest wall motion and results in decreased lung volume. Additionally, these changes occur at different times and stages of lung development after birth. TIS is usually progressive when the spinal chest wall deformity as well as the underlying muscle disease worsen. Progression of TIS is evaluated separately based on selected structural features (e.g. the Cobb angle) and selected functional features (e.g. lung function). Changes in spinal thoracic deformity and respiratory function are not consistent and do not appear to be correlated over time [79]. More comprehensive measurements of the 3D chest structures and thorough measurements of cardiorespiratory fitness may provide additional insight. Notably, some indicators of abnormal lung function, such as hypercapnia and pulmonary hypertension, may not appear unless the deformity is severe.

Most patients with spinal thoracic deformities exhibit clinical evidence of restrictive respiratory disease. The earliest manifestation is exercise-related shortness of breath, which is often considered (by the patient’s family) to be exercise intolerance or fatigue due to exertion. In one paediatric study of children under 10 years of age, the spirometry results showed that dyspnoea did not occur frequently until the FVC fell below 50%; thus, children with restrictive lung disease often adapt by reducing the intensity of their daily activities [80]. Some patients are sedentary to avoid breathing difficulties with activity. Restrictive changes can be measured either as a reduction in FVC at normal FEV1 or by measuring total lung volume via spirometry. Restrictive respiratory mechanics are caused by reduced chest wall compliance due to deformity, possibly due to reduced lung compliance resulting from decreased lung volume in different lung regions [80].

Hypoxemia and pulmonary hypertension have been reported in some TIS patients, and it is usually accompanied by hypercapnia respiratory failure, unless the patient is receiving oxygen therapy or non-invasive positive pressure ventilation [81]. Although there are no published data on the incidence of pulmonary hypertension in patients with TIS, there are data on the incidence of scoliosis showing that 38% and 31% of such patients died from respiratory failure and cardiovascular disease, respectively, for which pulmonary hypertension might have been the main cause [82].

## Histological changes

To examine the lungs, Japanese scholars performed an autopsy of an elderly TIS patient with scoliosis who had not been surgically treated [83]; the results revealed that the spine was curved to the left, the left lung was significantly flattened and the right lung was enlarged. From histological examination, there were no alveoli around the left upper lobe, which was a result of pulmonary constriction due to hemithoracic shortening caused by rib fusion. We suggest that physicians should encourage VEPTR treatment in infants and adolescents with TIS to reduce the risk of restricted lung development. Compared with the left lung, the right upper lobe exhibited obvious alveolar space with severe inflammatory cell infiltration. In animal model experiments of scoliosis [78, 84–86], the alveolar wall of rabbit lung tissue exhibited significant thickening, the number of alveoli decreased, and the cavity volume increased. The mechanism underlying volume loss was constriction of the lung due　to shortening of the hemithorax secondary to rib fusion. We propose that physicians should encourage VEPTR treatment in infant and juvenile TIS patients to decrease the future risk of pulmonary death.

## Clinical assessment

### Clinical manifestations

Worsening of deformity during growth is reflected as an increase in respiratory rhythm, resulting in dyspnoea and difficulty in performing daily activities [2]. TIS progressively worsens with the spinal thoracic deformity and underlying muscle condition. However, clinical symptoms are not consistent or correlated with changes in deformity or respiratory function over time [9]. This is why change in lung function is not observed until there is a significant reduction in chest expansion. The clinical symptoms of these patients are based on their easy fatigue, inability to perform daily activities and/or a history of many episodes of pneumonia or bronchitis [87].

### Physical examination

Physical examination assessments include evaluations of the respiratory rate, lung auscultation and body measurements (e.g. weight, height, chest circumference and extremity length). An abnormal respiratory rate indicates latent respiratory failure [9]. Lips and fingernail beds should be examined for cyanotic characteristics, as these may indicate chronic hypoxemia [9]. The finger excursion test should be performed to assess the loss of chest expansion capacity during breathing [4], and the Adams test to assess the presence of a rib hump caused by stooping conditions, and the general appearance of the back, flexibility of the spine, flexion and extension activities, rotation activities and other characteristics [9, 19].

### Radiological assessment

Imaging studies should include standing anteroposterior and lateral radiographs of the entire spine, as well as evaluations of the Cobb angle, thoracic vertebrae height and space available for the lungs on X-rays [19]. Lateral radiographs provide information on the sagittal depth of the chest due to pectus excavatum or thoracic lordosis. Radiographic findings (such as the spine length and/or chest size) are often reported as surrogate measures of lung function but cannot accurately describe or be correlated with lung function results [88].

CT scans of the thorax, abdomen and spine can confirm a severe loss of hemithoracic volume. In recent years, research has focused on the use of CT to estimate changes in lung volume [89]. However, because the lower edge of the lung moves during breathing, the values obtained may be less accurate [4]. Four-dimensional CT can depict chest movements, but it has the disadvantage of radiation exposure. Studies have shown that children managed for TIS using consistent protocols receive an average of four times the estimated average background radiation dose of 3 mSv/year in the USA. CT scans account for 74% of the total dose [90]. In fact, studies have indicated an increased incidence of cancer in patients with IS due to the need for multiple X-rays [90].

### Quantitative dynamic MRI assessment

All patients should undergo MRI of the entire spinal cord to rule out spinal cord abnormalities caused by twisting of the thoracic spine. The study shows that the high incidence of intraspinal anomalies in presumed idiopathic scoliosis emphasizes the need for detailed examination for subtle neurological signs that accompany neuro-axial anomalies. Preoperative MRI screening is recommended in patients with presumed ‘idiopathic’ scoliosis who present at young age, with neurological findings and in curves with apical thoracic kyphosis [91]. Current research is focused on developing a method to reduce the use of radiation-based imaging by developing more direct lung function tests such as lung MRI or other techniques [90].

MRI offers several advantages over CT [92]. Recent advances in MRI technology have improved the assessment of lung tissue and lung function. Intravenous contrast media for visualization of the pulmonary vasculature or lung perfusion, as well as inhalation of contrast media (nebulized gadolinium chelate, high concentrations of oxygen or inert gases to visualize air spaces), have significantly expanded the use of MRI in the assessment of lung function. Other lung function-imaging methods have been developed, including MRI of respiratory mechanics, diaphragm and chest wall motion and static and dynamic lung volumes. Dynamic MRI (dMRI) allows for spontaneous free breathing without the need for patient breath-holding or the wearing of a gated signal-acquisition device. Thus, dMRI enables the analysis of changes in lung volume attributable to bilateral chest wall and diaphragm displacement [93, 94]. By quantifying these displacements, the overall mechanical function of the thoracic cavity can be assessed based on the structure of the spine, ribs and implanted devices before and after surgery; hence, dMRI is the most appropriate imaging modality for patients with TIS.

Tong et al. [95–97] describes a quantitative MRI method for comparing regional dynamics of the lung, chest wall and hemidiaphragm and changes in local dynamic chest function before and after surgical treatment in children with TIS. The mean left and right lung volumes at end-inspiration and end-expiration were found to increase significantly postoperatively, and the mean lung tidal volumes also improved, with no change from conventional pulmonary function tests. Quantitative dMRI may, for the first time, precisely quantify thoracic spinal deformities and abnormal dynamics that appeared before and after surgical treatment, providing a new scientific understanding of the biomechanical basis of EOS and a new objective indicator of EOS treatment response. Therefore, a routine MRI assessment in this population may be recommended to capture chest and lung changes early on and potential prevent or delay progression of symptoms.

### Assessment of pulmonary function

Pulmonary function is one of the most important functional outcomes of TIS. Although the most reliable method of lung function assessment is probably the preoperative and postoperative pulmonary function test (PFT). However, there are real challenges in assessing pulmonary function in children 2–6 years of age because they are unable to sedate and have difficulty cooperating with all breathing manoeuvers. These challenges can be greatly improved by team experience and motivation to improve the success rate of test data. Devices that use resistance measurements (forced oscillation technique, intermittent apparatus technique and volumetric tracings) and spirometry in clinical settings can monitor treatment and lung development in congenital and early acquired respiratory diseases [98]. Pulmonary dysfunction in children with TIS is characterized by a reduced chest volume, decreased residual capacity and lower vital capacity [79]. Some patients, due to increase residual capacity, are unable to fully exhale, therefore, further reducing FVC.

The impact of restrictive pulmonary dysfunction on lung function should be examined by assessing arterial blood gases and PFT results [99]. PFTs cannot be used effectively in patients under 5 years of age, because these tests require forced exhalation and inhalation, and patients at this age cannot cooperate well. However, some scholars can complete the lung function test by letting children play computer-incentive games. In younger children, these measures of lung function can be passively determined using external pressure under general anaesthesia [80, 87, 100]. However, sedation methods do not take into account the effects of accessory muscles during breathing [9].

### Haemoglobin

Elevated haemoglobin (Hb) is associated with chronic hypoxia and may be a surrogate indicator of preoperative disease severity in TIS patients. Previous retrospective studies have noted a decrease in Hb in EOS patients with high Hb within 6 months after thoracoplasty or growth rod (GR) surgery [101, 102]. Elevated Hb levels may be a marker of disease severity and can be ameliorated by GR techniques. In patients with EOS presenting with TIS, Hb tests may be a suitable substitute for PFTs and CT.

### Other

The study of pulse oxygen saturation is helpful for detecting hypoxia. When there are problems with early cor pulmonale, echocardiography is used to detect tricuspid regurgitation and pulmonary hypertension.

## Clinical treatment

Spine and chest wall reconstructive surgery may be necessary in children with EOS when progressive EOS limits lung development and chest function. Expansion thoracoplasty (ET) for severe TIS in patients with severe juvenile spinal deformity improves and stabilizes respiratory function [103].

ET was performed on a juvenile rabbits with EOS, and its effects on thoracic development and respiratory function were quantified [62]. Early- and late-treated rabbits were compared to age-matched normal (disease-free) control rabbits until skeletal maturity. Early and late ET improved spinal deformities, lung growth inhibition and respiratory dysfunction in the rabbits; notably, early treatment appeared to block the natural progression of scoliosis-related deformities and pulmonary dysplasia in rabbits [84].

**Fig. 3:**
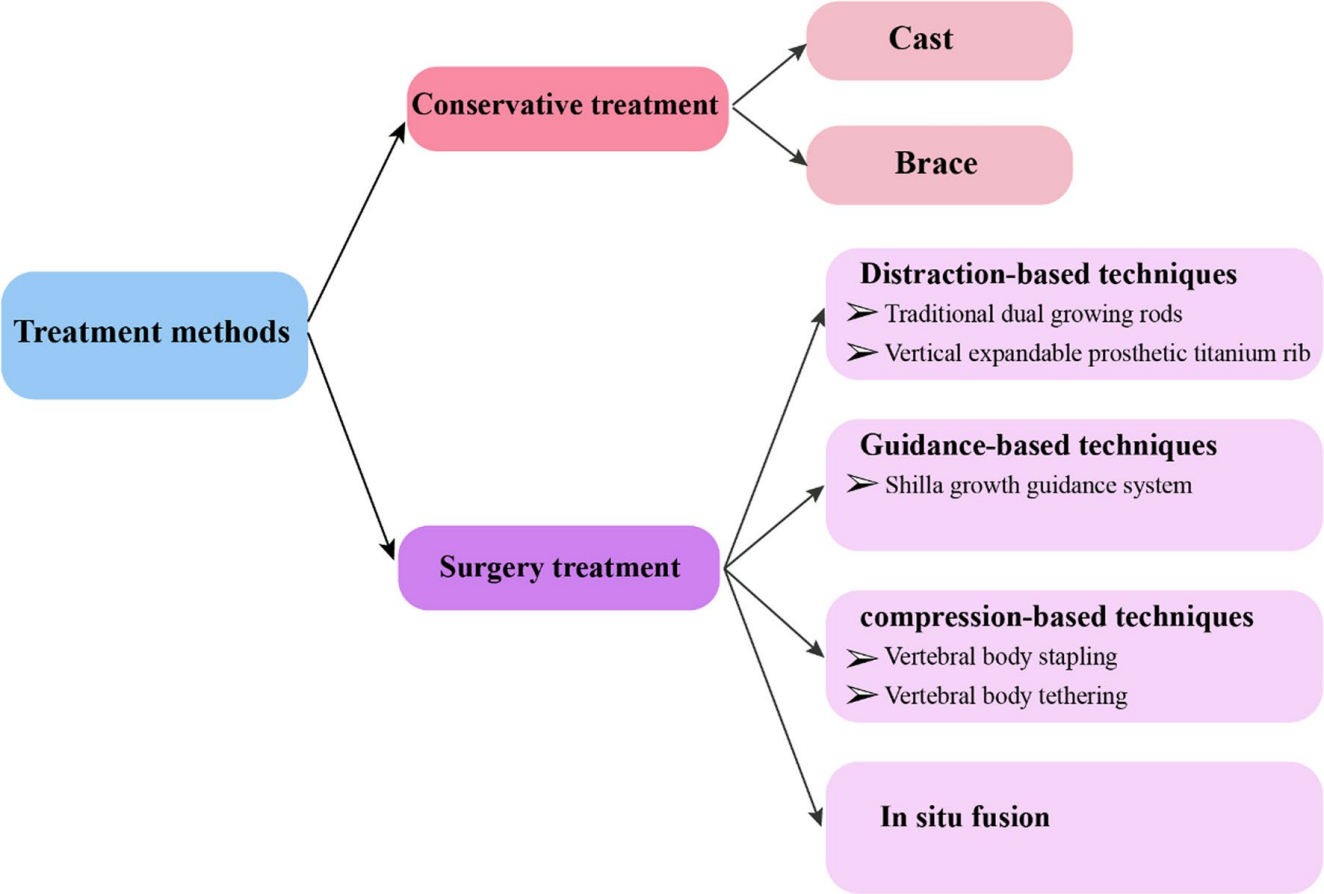
Classification of EOS treatments. EOS = early-onset scoliosis

Growth-preserving spinal and thoracic stabilization strategies are available with a number of tools, including braces, casts, GRs, the vertical expandable prosthetic titanium rib (VEPTR) and MAGnetic Expansion Control (MAGEC) rods (Fig. 3). The goal of any growth-promoting surgical strategy is to modify the natural history of associated cardiorespiratory deficits and the progression of underlying spinal deformities or negative changes in spinal biomechanics due to implant fixation [104].

### Conservative treatment

#### Casts

Due to the adverse natural history of untreated infantile IS, the task of spine surgeons is to identify patients who are likely to progress and to treat these children in a way that promotes lung development and stops scoliosis progression. The application of serial casts in the Mehta manner can stop or limit scoliosis progression and, in some children, correct scoliosis over the long term [105]. Currently, the Mehta derotation series casts are an important part of the treatment, not only for infantile IS but also for syndromic and congenital patients. Studies have shown that conservative treatment can effectively delay surgery and reduce the incidence of complications due to surgery [106].

#### Braces

Bracing is considered to be the traditional method of controlling EOS. However, it has limited effect on the treatment of neuromuscular scoliosis values in children and does not prevent the development and progression of scoliosis in this population. Mehta et al. [107] showed that in children with scoliosis complicated by spinal cord injury, as curve size increases (> or = 20 degrees), bracing seems to play a limited role, because it does not seem to prevent surgery or delay time to surgical correction. In infantile IS, the function of the brace is primarily to stabilize rather than ameliorate the deformity; thus, the brace is less likely to result in permanent correction of scoliosis as opposed to a serial cast. It is also indicated for patients with progressive scoliosis and an intolerance to casts, as well as those for whom surgical treatment is contraindicated for medical or other reasons [108]. For children with scoliosis of more than 35°, a brace can be considered successful if disease progression is stopped within a few years.

### Surgery

TIS is a progressive disease that limits the growth of the thoracic spine and lungs. Treatment options vary from focusing on fusion and spinal stabilization to spinal stabilization and growth [109]. The timing and type of surgical treatment depend on the disease itself, the associated morbidity and the expected progression and prognosis.

#### Spinal fusion

Although early spinal fusion can stop progression in children with EOS, it results in spinal and thoracic growth inhibition and limited lung growth, which potentially lead to TIS. Patients with spondylothoracic deformities are at the highest risk of developing TIS if more than four levels of vertebral bodies are fused, especially those with rib abnormalities [11, 110]. If the deformity is confined to a few segments and growth of the remaining spine is still allowed, limited arthrodesis or resection of the apical region may be performed. There are also patients who achieve sufficient spinal length and thoracic volume to allow for the progression of the deformity to achieve permanent correction, for whom final spinal fusion is appropriate. The timing of this surgery is controversial, but in general, patients who are at least 10 years of age and who have completed most of their thoracic growth are considered good candidates for final fusion to complete spinal deformity treatment [111].

#### Vertical expandable prosthetic titanium rib (VEPTR)

Robert Campbell designed the VEPTR in 1989 as a non-fusion, growth-friendly implant for patients with severe EOS [112]. It requires a specific surgical strategy based on volume-reducing deformities to guide the management of dilated thoracoplasty [3]. Because the VEPTR can specifically target chest wall growth, it has become a common treatment modality for patients with scoliosis and rib fusion [113, 114]. The goal of this device is to achieve sufficient chest height to optimize lung volume and promote lung development. There is a relatively high rate of reported complications with VEPTR in the literature. The potential complications of VEPTR include infection, rib fracture, dislodged hardware and neurological injury [115].

#### Traditional and magnetic growth rods (GRs)

Growth-friendly surgery for the treatment of young children with EOS has been used for more than half a century, following an early approach involving distraction surgery without fusion [116]. This technique was later refined to the placement of rods and hooks through subcutaneous sites. Individual treatment of a group of EOS patients with a single submuscular GR was reported in 2001 [117]. Dual GR technology, with which GRs can be placed submuscularly or subcutaneously, provides better stability and better correction of lateral curvature [118–120]. Continued improvements in technology have allowed for better initial correction and maintenance of correction throughout the treatment process, minimizing the risk of spontaneous fusion and successfully preventing the development of scoliosis by maintaining spinal growth.

The biggest technological shift came with the introduction of magnetic controlled GRs (MCGRs). The MCGR non-invasively stretches the spine using an external remote control in an outpatient setting, allowing for periodic non-invasive lengthening without the need for open surgery. The safety and efficacy of MCGRs for successful correction of spinal deformities have been reported in many cases [121]. Additionally, this technique is effective with respect to minimizing the negative effects of repeated surgeries and anaesthesia events, especially in very young children. Moreover, treatment with MCGRs has produced results similar to those for treatment with double GRs in terms of scoliosis correction and growth while allowing for non-invasive lengthening, thus reducing the number of planned surgeries and the complication rates [122].

#### SHILLA growth guidance system

In the SHILLA growth guidance system (SGGS) procedure [123], multiplanar correction is achieved via pedicle fixation and fusion in the deformed apical region, including local fusion using non-locking polyaxial screws proximally and distally, followed by guiding a rod that remains in place. A certain length is necessary to minimize the need for subsequent surgery. Although more segments are fixed compared to conventional GRs, deformity control may be corrected with sliding screws. However, at the upper end, this can lead to soft tissue irritation; at the lower end, it can interfere with the lumbar facet joints. In a retrospective multicentre study, treatment using the SGGS led to significantly fewer overall procedures but more unplanned procedures due to implant complications compared with GRs [124].

#### Compression-based techniques (stapling and tethering)

Thoracoscopic placement of memory metal stapling or flexible tethering to guide anterior growth can also be used to avoid repeat surgeries. This approach leaves the posterior structures of the spine unaffected and is not dependent on critical soft tissue coverage. The principle of applying convex side compression to the growth zone of the vertebral body by spanning the intervertebral disc is thought to result in deformity-corrected asymmetric growth. This effect appears to be limited to patients with good flexibility and mild scoliosis (< 35°) with a success rate (partially corrected or stabilized) of 70–80%, comparable to traditional braces [125]. The use of flexible tethers to interconnect transverse vertebral screws is less common in clinical practice. To date, data on the efficacy and safety in patients with EOS are lacking [126].

## Conclusion

TIS in children with early-onset scoliosis manifests as a history of prominent respiratory symptoms, chest deformity on physical examination, abnormal chest X-ray and CT findings and changes in pulmonary function. A potential therapeutic goal for this syndrome is to restore chest function and volume during growth. Although some patients exhibit good spine growth with growth-friendly surgery, there is still a subset of patients with poorer lung outcomes in adulthood. Future research must continue to focus on classification results based on the specific diagnoses of EOS patients. Due to the repetitive nature of prolonged surgery, treatment remains challenging and complication rates are high. Accordingly, more research is needed to further reduce complications and improve outcomes after EOS. Successful treatments that encourage the growth of the spine and chest will lead to good outcomes for EOS patients. With the increase of curvature, EOS patients can lead to structural changes in the chest cavity, causing severe complications such as restrictive lung disease, cardiovascular complications and respiratory failure. However, the treatment of children with EOS is customized according to specific diseases. Although lack of treatment has been proved to lead to an increase in mortality, extensive early and definite fusion may lead to thoracic insufficiency. Delaying definite surgery and using increasing instruments may be beneficial to keeping lungs healthy.

## Data Availability

Not applicable.
